# Discrepancy Between Invasive and Noninvasive Blood Pressure Measurements in Patients with Sepsis by Vasopressor Status

**DOI:** 10.5811/westjem.2022.1.53211

**Published:** 2022-05-05

**Authors:** Quincy K. Tran, Dominique Gelmann, Zain Alam, Richa Beher, Emily Engelbrecht-Wiggans, Matthew Fairchild, Emily Hart, Grace Hollis, Allison Karwoski, Jamie Palmer, Alison Raffman, Daniel J. Haase

**Affiliations:** * University of Maryland School of Medicine, The R Adams Cowley Shock Trauma Center, Baltimore, Maryland; † University of Maryland School of Medicine, Department of Emergency Medicine, The Research Associate Program in Emergency Medicine and Critical Care, Baltimore, Maryland

## Abstract

**Introduction:**

Blood pressure (BP) monitoring is an essential component of sepsis management. The Surviving Sepsis Guidelines recommend invasive arterial BP (IABP) monitoring, although the benefits over non-invasive BP (NIBP) monitoring are unclear. This study investigated discrepancies between IABP and NIBP measurement and their clinical significance. We hypothesized that IABP monitoring would be associated with changes in management among patients with sepsis requiring vasopressors.

**Methods:**

We performed a retrospective study of adult patients admitted to the critical care resuscitation unit at a quaternary medical center between January 1–December 31, 2017. We included patients with sepsis conditions AND IABP monitoring. We defined a clinically significant BP discrepancy (BPD) between NIBP and IABP measurement as a difference of > 10 millimeters of mercury (mm Hg) AND change of BP management to maintain mean arterial pressure ≥ 65 mm Hg.

**Results:**

We analyzed 127 patients. Among 57 (45%) requiring vasopressors, 9 (16%) patients had a clinically significant BPD vs 2 patients (3% odds ratio [OR] 6.4; 95% CI: 1.2–30; P = 0.01) without vasopressors. In multivariable logistic regression, higher Sequential Organ Failure Assessment (SOFA) score (OR 1.33; 95% CI: 1.02–1.73; P = 0.03) and serum lactate (OR 1.27; 95% CI: 1.003–1.60, P = 0.04) were associated with increased likelihood of clinically significant BPD. There were no complications (95% CI: 0–0.02) from arterial catheter insertions.

**Conclusion:**

Among our population of septic patients, the use of vasopressors was associated with increased odds of a clinically significant blood pressure discrepancy between IABP and NIBP measurement. Additionally, higher SOFA score and serum lactate were associated with higher likelihood of clinically significant blood pressure discrepancy. Further studies are needed to confirm our observations and investigate the benefits vs the risk of harm of IABP monitoring in patients with sepsis.

## INTRODUCTION

Sepsis and septic shock are major healthcare problems that affect millions of people around the world annually.^1^ The Surviving Sepsis Campaign (SSC) guidelines suggest maintaining a mean arterial pressure (MAP) of at least 65 millimeters of mercury (mm Hg) among these patients.^1^ Furthermore, a recent meta-analysis suggested that earlier administration of vasopressors is associated with improved short-term outcomes in patients with sepsis.^2^ Invasive arterial blood pressure (IABP) monitoring is considered to be the gold standard compared to non-invasive blood pressure (NIBP) monitoring.^3^ Despite the SSC recommendation that patients requiring vasopressors should undergo arterial catheter placement for IABP monitoring as soon as possible,^1^ only 52% of patients on vasopressors from 168 intensive care units (ICU) across the United States had IABP monitoring.^4^

Data regarding the efficacy of IABP monitoring has been inconclusive. A previous retrospective study of 30 patients with septic shock^5^ suggested there was only a small difference in MAP measurements between IABP and NIBP in its small patient population, and only 10% of those patients had a difference of ≥ 10 mm Hg. However, the study was significantly limited by its small patient sample size and lack of control group. More importantly, the study did not assess whether having IABP monitoring would have changed patient management compared to NIBP monitoring.

In our study we investigated the discrepancy between NIBP and IABP measurement in a large patient population with septic shock defined by the use of vasopressors, compared to a control group of patients with sepsis but without vasopressors. We hypothesized that the use of vasopressors would be associated with an increased discrepancy between NIBP and IABP measurement, which would translate into potential differences in clinical management for patients with septic shock.

## METHODS

### Study Setting

We conducted the study in the critical care resuscitation unit (CCRU) at a quaternary academic center. The goal of the CCRU (created in July 2013) is to expedite the interhospital transfer of patients with time-sensitive disease or critical illnesses when these conditions exceed the capability of the referring hospitals and when our medical center’s adult ICUs do not have an available bed.^6^ These patients, depending on their disease severity, are transferred urgently to the CCRU to undergo diagnostic or therapeutic interventions. Once these patients receive the necessary interventions and are stabilized, they are moved to an available in-patient bed at our medical center.

To resuscitate these patients in the acute phase, the CCRU clinical policy requires that patients have arterial blood pressure monitoring if they need frequent blood gas analyses or hemodynamic monitoring, whether receiving a vasoactive infusion or not. The CCRU nursing staff also document hourly BP measurements. Patients who do not need further ICU level of care can have BP recorded every 2–4 hours while waiting in the CCRU for a bed in an intermediate care (IMC) unit or medical ward. Most of the arterial catheter cannulations are performed by CCRU clinicians upon patients’ arrival as part of the resuscitation efforts. The cannulations are performed under sterile conditions with sterile gloves, sterile fields, and hair covers in compliance with our institutional requirements. Additionally, the cannulation process can be aided by point-of-care ultrasound at the clinicians’ preference. Our study was approved by our institutional review board.

**Population Health Research Capsule**

What do we already know about this issue?

*There are discrepancies in invasive arterial blood pressure (IABP) and non-invasive BP (NIBP) measurements in patients with sepsis.*

What was the research question?

*Does the difference between IABP and NIBP lead to change in management among patients with septic shock.*

What was the major finding of the study?

*Vasopressor use, Sequential Organ Failure Assessment (SOFA) score, and lactate levels are associated with change in management between IABP and NIBP monitoring. NIBP was typically higher than IABP.*

How does this improve population health?

*Invasive arterial blood pressure monitoring is associated with detection of occult hypotension, compared to NIBP, in septic patients with shock, high SOFA score, or high lactate level.*

### Patient Selection

This study is a secondary analysis of a previously collected clinical dataset.^7^ All adults who were admitted to the CCRU between January 1–December 31, 2017 with arterial catheter cannulation at the CCRU were eligible. We included patients with diagnoses suggesting sepsis conditions and NIBP and IABP measurement within 60 minutes of each other. We excluded patients who had diagnoses of hypertensive emergencies (acute aortic diseases, spontaneous intracranial hemorrhage, ischemic stroke, etc.) because these patients are managed according to goals of systolic BP,^8^,^9^ while patients with sepsis are managed according to goals of MAP.^10^ We also excluded patients who did not have three BP measurements for each modality (IABP and NIBP) because we suspected that a lower number of BP measurements would not produce reliable average values of the measurements. Patients who arrived at the CCRU with arterial catheters were also excluded because they would not have documentations of IABP measurements at the time of arterial catheter insertions. We defined patients with shock as those requiring any vasopressor (eg, norepinephrine, epinephrine, vasopressin) as reported previously.^5^ For our study, we included only patients who received vasopressors within one hour of arterial cannulation.

### Data Collection and Management

We collected data from patients’ electronic health records at our institution. Relevant data occurring within one hour of arterial cannulation was collected retrospectively. Demographic data included age, gender, past medical history, and body mass index. Clinical data included components of the Sequential Organ Failure Assessment (SOFA) score, white blood cell (WBC) counts, serum lactate levels, and four consecutive pairs of both IABP and NIBP measurements. For components of the SOFA score, we imputed missing components as normal. Three patients in our population did not have laboratory values for total bilirubin at the time of arterial cannulation. Because their values were normal at subsequent laboratory checks we imputed their component for the liver SOFA score as normal (score of 0). We also extracted data regarding complications from arterial catheter insertions throughout a patient’s hospital stay. We defined complications as any necrosis of hand, wrist or extremity, source of blood stream infection or local infection, bleeding, or aneurysm.

We performed our retrospective data analyses in compliance with methodologic standards for health record review.^11^ The research team members, who were not blinded to the study hypothesis, were first trained by the principal investigator to extract data into a standardized Excel spreadsheet (Microsoft Corp, Redmond, WA). Training was performed with sets of 10 patients until results from all research team members reached 90% agreement with a senior investigator. Up to 10% of each investigator’s data was subsequently double-checked for accuracy. To reduce further bias, investigators independently collected data in separate sections. For example, investigators who collected data for SOFA scores did not collect BP measurements, and vice versa.

### Outcome Measures

Our primary outcome was the percentage of patients who had a clinically significant BP discrepancy (BPD) in MAP measurement via IABP and NIBP between those receiving vasopressors and those not receiving vasopressors. We defined a clinically significant BPD as a difference of at least 10 mm Hg AND a potential change of clinical management, according to patient’s goal MAP ≥ 65 mm Hg. For example, when the MAP from a patient’s arterial catheter was 58 mm Hg but the MAP from NIBP was 68 mm Hg, this was considered a clinically significant BPD. In this case, crystalloids or even vasopressors would have been added to increase the patient’s MAP of 58 mm Hg, while the MAP of 68 mm Hg, according to NIBP monitoring, would have suggested no further interventions. Conversely, a patient with a MAP of 50 mm Hg per IABP and MAP of 60 mm Hg per NIBP would not have a clinically significant BPD, because both modalities would have suggested interventions to increase the MAP to reach a goal of 65 mm Hg.

Our secondary outcome was the percentage of patients who had MAP differences between IABP and NIBP of at least 10 mm Hg. Other outcomes included factors associated with either primary or secondary outcomes.

### Sample Size Calculation

We based our sample size calculation on previous results by Riley et al.^5^ We planned to detect a difference of 10 mm Hg with a standard deviation of 15 between NIBP-IABP among patients with vasopressors and those without vasopressors. As a result, we calculated that we would need 37 patients for each group to have power of 80% with an α value of 0.05.

### Data Analysis

We used descriptive analyses (mean ± standard deviation [SD]), median [interquartile range [IQR]), or percentages to present continuous variables or categorical variables as appropriate. We used unpaired Student’s t-test to compare the mean between two groups (without vs with vasopressors). We performed forward stepwise, multivariable logistic regressions to estimate the associations between demographic, clinical independent variables with our outcomes (clinically significant BPD, MAP difference ≥ 10). Our independent variables were determined a priori and are listed in Appendix 1. Additionally, we assessed the goodness-of-fit, multicollinearity, and discriminatory capability of our multivariable logistic regression models. For goodness-of-fit tests, a model with Hosmer-Lemeshow test’s *P*-value > 0.05 is considered to have a good fit of independent variables.

We used variance inflation factors (VIF) to assess independent variables’ multicollinearity. Any factor with VIF ≥ 5 were removed from the logistic regression for demonstrating collinearity. We used the area under the receiver operating characteristic (AUROC) curve to assess our logistic regression models’ discriminatory capability. A model with AUROC of 1.0 would be considered to have perfect discriminatory capability because this model can perfectly distinguish the difference between dichotomous outcomes (eg, clinically significant BPD vs none), while a model with AUROC of 0.5 would have poor discriminatory capability.

### Additional Analyses

Once our multivariable logistic regression identified continuous independent variables that were significantly associated with a clinically significant BP discrepancy in IABP and NIBP measurement between patients with and without vasopressors, we applied those continuous independent variables in probit analyses. The probit analyses would enable us to predict the probability of clinically significant BPD at certain values of the continuous independent variables. We used the Bland-Altman plot to graphically present the discrepancy between NIBP and IABP. We performed our statistical analyses with Minitab version 19 (Minitab Corp, State College, PA). We considered all tests with two-tailed *P*-value < 0.05 as statistically significant.

## RESULTS

### Patient Characteristics

**Figure 1:**
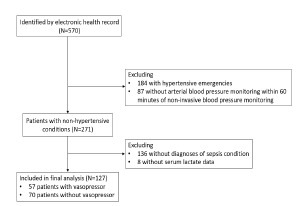
Patient selection diagram. We included 127 patients with sepsis conditions in our analysis.

We electronically identified 570 patients who underwent arterial catheter placement at the CCRU during the study period (Figure 1). Among 271 patients with non-hypertensive conditions, we included 127 patients with sepsis conditions (list of diagnoses is included in Appendix 2) based on their admission diagnoses. Among the included patients, 57 (45%) required vasopressors and 70 (55%) did not require vasopressors (Table 1).

The average (SD) age for the population was 55 (16) years (Table 1), and there was no age difference between patients without vasopressors or those with vasopressors. Compared to those without vasopressor use, patients who required vasopressors had significantly higher WBC counts, serum lactate levels, and SOFA scores (Table 1). Other clinical factors were similar between both groups. Of the patients requiring vasopressors, 19 (33%) had MAP of less than or equal to 64 mm Hg by IABP monitoring, compared to 6 (9%) of those without vasopressors (OR 5.3; 95% CI:1.9–14.5; *P*< 0.001) (Table 2). In other words, IABP monitoring was associated with a 5.3-times higher likelihood of detecting MAP level less than the recommended level of 65 mm Hg in sepsis patients requiring vasopressors.

The median IQR of catheter days was 3 (1–5). The total number of catheter days for our patient population was 639, with no complications (95% CI: 0–0.02) from arterial catheter insertion (Table 2).

### Primary Outcome: Clinically Significant Discrepancy Between NIBP And IABP

Among 57 patients requiring vasopressors, nine patients (16%) had a clinically significant BP discrepancy, compared to two patients (3%) without vasopressor requirement (OR 6.4; 95% CI: 1.2–30; *P* = 0.01) (Table 2).

**Figure 2:**
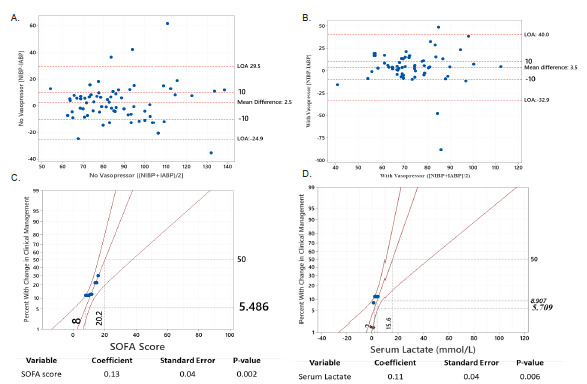
(A)Bland-Altman plot displaying blood pressure differences among septic patients without vasopressors. The noninvasive blood pressure (NIBP) and invasive arterial (IA) BP discrepancy was distributed evenly throughout the X-axis, demonstrating that the difference between the two modalities occurred when patients were hypotensive or normotensive. Additionally, the difference between NIBP and IABP on the Y-axis was mostly concentrated between the level of −10 mm Hg and +10 mm Hg, demonstrating that the NIBP modality has equal likelihood to be higher or lower than IABP. (B) Bland-Altman plot displaying blood pressure differences among septic patients with vasopressors. There were even distributions of NIBP-IABP* discrepancies along the X-axis, demonstrating that the difference between the two modalities occurred when patients were hypotensive or normotensive. However, most values for [NIBP-IABP] difference were above the level of +10 mm Hg, demonstrating that NIBP measurements were usually greater than IABP in our patient population with sepsis requiring vasopressors. (C) Probit logit analysis showing probability of having clinically significant discrepancy between noninvasive and intra-arterial blood pressure (Y-axis) and its association with SOFA score (X-axis). Patients who had a SOFA* score of 20 (X-axis) would have 50% probability (Y-axis) of requiring change in clinical management when arterial catheters were inserted. (D) Probit logit analysis showing probability of having a clinically significant discrepancy between noninvasive and intra-arterial blood pressure (Y-axis) and its association with serum lactate level. Patients who had serum lactate of 4 mmol/L (X-axis) would be associated with approximately 9% probability (Y-axis) of having change of clinical management when arterial catheters were present. *IABP*, invasive arterial blood pressure; *LOA*, limit of agreement; *mm Hg*, millimeter of mercury; *NIBP*, non-invasive blood pressure; *SOFA*, Sequential Organ Failure Assessment; *mmol/L*, millimoles per liter.

The Bland-Altman plot of patients with sepsis but not requiring vasopressors (Figure 2A) showed that the [NIBP-IABP] discrepancy was distributed evenly throughout the X-axis, which suggested that the difference between the two modalities was distributed evenly when patients were hypotensive or normotensive. Additionally, in this patient population, the discrepancy between NIBP and IABP (denoted as [NIBP-IABP] on the Y-axis) was mostly concentrated between the level of −10 mm Hg (IABP measurements > NIBP measurements) and level of +10 mm Hg (NIBP measurements > IABP measurements) (Figure 2A). This distribution suggested that there was similar likelihood for IABP to be higher than NIBP, and vice versa, among patients with sepsis not requiring vasopressors.

Among patients with sepsis requiring vasopressors, the NIBP and IABP difference was also distributed evenly along the X-axis (Figure 2B). However, most values for the NIBP and IABP difference for this group were above the level of +10, suggesting that NIBP measurements were in general greater than IABP in patients with sepsis requiring vasopressors.

Table 3 shows the results of the multivariable logistic regressions measuring the association between clinical factors and the primary outcome of clinically significant BP discrepancy between NIBP and IABP measurement. Four factors were associated with a clinically significant BPD between NIBP and IABP. Each unit increase in SOFA score was associated with increased odds of having a clinically significant difference in management when comparing NIBP and IABP (OR 1.33; 95% CI: 1.02–1.73; *P* = 0.034). Similarly, each increase in millimoles per liter (mmol/L) of serum lactate was associated with increased odds of having a clinically significant BP discrepancy when an arterial catheter was inserted (OR 1.27; CI: 1.003–1.60; *P* = 0.047). The model showed good fit of data (Homes-Lemeshow test’s *P* = 0.81), low multicollinearity (all factors had VIF < 5), and very good discriminatory capability (AUROC = 0.92).

Probit logit analyses demonstrated that for patients with a mean SOFA score of 8 (approximately 5% of all patients with sepsis, regardless of vasopressor status) had a clinically significant BP discrepancy causing change in management when an arterial catheter was inserted (Figure 2C). Similarly, when a patient’s serum lactate level was 2 mmol/L (approximately 6% of all patients with sepsis, regardless of vasopressor status), IABP monitoring resulted in a change in clinical management (Figure 2D). Approximately 9% of patients had a change in clinical management when their serum lactate was 4 mmol/L.

### Secondary Outcome: MAP Difference ≥ 10 mmHg Between NIBP And IABP

Three factors were significantly associated with high likelihood of patients having a MAP difference ≥ 10 mm Hg between the two modalities (Table 3). These three factors were higher SOFA score (OR 1.27; 95% CI: 1.03–1.3; *P* = 0.012), having peripheral artery disease (OR 6.7; 95% CI: 1.3–22.5; *P* = 0.021), and the diagnosis of incarcerated organs (OR 16.4; 95% CI: 1.4 to +100; *P* = 0.027).

## DISCUSSION

The use of vasopressors was associated with an increased incidence of clinically significant BP discrepancy between IABP and NIBP in our population of patients with sepsis. Among all patients with sepsis (both those requiring vasopressors and those not on vasopressors), a few clinical factors were associated with increased odds of change in BP management when arterial catheters were inserted. Higher SOFA score and higher serum lactate levels were both associated with higher likelihood of clinically significant BP discrepancy between NIBP and IABP monitoring modalities. Higher SOFA score, history of peripheral artery disease, and the diagnosis of incarcerated organs were associated with higher likelihood of larger MAP difference between NIBP and IABP.

Our study provides support for the use of IABP monitoring in patients requiring vasopressors, as clinically significant BP changes may be missed with NIBP monitoring. Findings from our study population showed that IABP monitoring in patients with sepsis requiring vasopressors was significantly associated with higher likelihood of detecting MAP ≤ 64 mm Hg (Table 2). Similarly, IABP values were more frequently lower than NIBP among patients with sepsis on vasopressors (Figure 2B). Although the mechanism for the difference is still unknown, this observation may have important clinical implications. Patients who require vasopressors may have unrecognized hypotension when they arrive at a resuscitation or ICU due to the use of NIBP monitoring. Additionally, NIBP measurements are typically taken intermittently, while IABP measurement provides the additional benefit of real-time continuous monitoring. For both reasons, IABP monitoring would enable clinicians to detect MAP < 65 mm Hg sooner and intervene early. This has important implications for patient outcomes, as previous studies suggested that hypotension may lead to significant morbidity for patients.^12^,^13^

In a patient population that is similar to ours, inserting an arterial catheter would be associated with a change in BP management in 9% of all patients with sepsis regardless of vasopressor status, and in 16% of patients with sepsis requiring vasopressors. In other words, for every 11 septic patients with IABP monitoring regardless of vasopressor use, one patient would be identified as requiring change in clinical management. For patients with sepsis requiring vasopressors, IABP monitoring would detect one need for change in management for every seven patients. Within our population of patients with sepsis, the probability of change in management when arterial catheter was inserted was approximately 5% for patients whose SOFA score was 8, for an estimated change in one of every 20 patients. For those with a SOFA score of 16, IABP monitoring was associated with management change in one of every two patients (Figure 2C). Our probit logit analysis suggested that IABP monitoring would detect one change in management for approximately every 17 patients with a serum lactate level of 2 mmol/L, and one change in management for every 11 patients with a serum lactate level of 4 mmol/L.

The baseline differences in patients with sepsis requiring vasopressors and those not requiring vasopressors may have influenced our findings of an increased rate of BP discrepancy in the former group. Patients with higher SOFA scores indicating increased illness severity may undergo more vasodilatory changes that may contribute to a higher prevalence of BP discrepancy between the two measurement modalities. While our patient population was not large enough for propensity score matching of these groups, further studies should explore such factors potentially influencing outcomes.

Although our study was not designed to investigate the economics of IABP monitoring, we calculated the cost required to detect change in clinical management via arterial line BP measurement for patients with septic shock. The one-time supply cost to set up IABP monitoring at our institution is approximately $55 US dollars (USD) per patient. The prevalence of clinically significant BP discrepancy was 16% among patients with sepsis on vasopressors, which equates to one change in management for approximately every seven patients with IABP monitoring. Therefore, the total cost of IABP monitoring would be approximately $385 USD to detect clinical change in management for every seven patients with sepsis requiring vasopressors. Further analysis is necessary to investigate whether the cost for IABP will offset the cost of patients’ hospitalization if they develop acute kidney injuries or other comorbidities.

Other authors have questioned the need for arterial catheters among critically ill patients because of the associated risks and unclear benefit of ABP.^14^ Results from our study suggest that IABP monitoring offers the benefit of potential change in clinical management due to early detection of hypotension, and with low cost and low complication rates. We found no complications from arterial catheter cannulation in our patient population. Our findings agreed with the previously reported low arterial cannulation risk of less than 1%.^15^ This suggests that early insertion of an arterial catheter is a low-risk procedure that enables clinicians to detect and remedy hypotension effectively, thus reducing the likelihood of hypotension-related complications. As a result, we presented information suggesting that arterial catheters are associated with a high benefit-to-harm ratio in patients with sepsis, especially those requiring vasopressors. Further studies are necessary to confirm our observations.

## LIMITATIONS

Our exploratory study has several limitations. First, patients with sepsis requiring vasopressors are not similar to those without vasopressors, but the small sample size prevented us from performing propensity score matching. However, by including the group of patients without vasopressors, we provided a glimpse of the potential discrepancy between NIBP and IABP in both groups of patients with sepsis. Additionally, we could not retrospectively identify whether the BP cuff was on the same or opposite arm as the arterial catheters, as our nursing staff usually does not document the location of the BP cuffs. Further, we based our outcome on the potential change of clinical management, according to MAP ≥ 65 mm Hg, but we could not ascertain what types of interventions were given to the patients. Finally, our multivariable logistic regressions showed wide 95% CIs for a few clinical factors (past medical history of kidney disease, diagnosis of bowel obstructions, and incarcerated organs) due to the very small sample sizes of these variables from a population with various causes of sepsis. Although our results suggest there may be an association between these factors and clinically significant BP discrepancy, the sample size was too small to draw meaningful conclusions.

Despite these limitations, our exploratory study had strength over the previous study by Riley et al.^5^ We included a larger number of patients requiring vasopressors along with a group of patients without vasopressors. We demonstrated relevant clinical benefits from IABP, not just the existence of discrepancy between NIBP and IABP measurements. Additionally, we identified a few clinical factors that may help clinicians practicing in the acute phase, such as in an emergency department, resuscitation unit, or ICU, to decide whether IABP is indicated.

## CONCLUSION

In patients with sepsis conditions requiring vasopressors, there was an increased likelihood of clinical change in blood pressure management with the use of invasive arterial blood pressure monitoring. There were no complications from arterial catheter insertion observed. Higher Sequential Organ Failure Assesment score and higher serum lactate levels were both associated with a higher likelihood of a blood pressure discrepancy leading to clinical change in management. Further studies are necessary to confirm our observation and investigate the risks of arterial catheter cannulations.

## Supplementary Information





## Figures and Tables

**Table 1:** Characteristics of patients with sepsis conditions and arterial pressure monitoring in the critical care resuscitation unit who were included in the study. Patients who required vasopressors were more likely to have higher SOFA* scores, serum lactate levels.

Variables	All patients (N = 127)	Without vasopressor (N = 70)	With vasopressor (N = 57)	P
Age, years (mean, SD)	55 (16)	54 (16)	56 (16)	0.3
Gender, N (%)				
Male	78 (61)	42 (60)	36 (63)	0.7
Female	49 (39)	28 (40)	21 (37)	0.7
BMI, mean (SD)	32.4 (11.9)	32.1 (10.7)	32.8 (13.3)	0.6
Past medical history, N (%)				
Diabetes	42 (33)	23 (33)	19 (33)	0.9
HTN	57 (45)	31 (44)	26 (46)	0.9
CAD	20 (16)	13 (19)	7 (12)	0.3
PAD	9 (7)	4 (6)	5 (9)	0.5
Any kidney disease	63 (50)	28 (40)	35 (61)	0.02
Mechanical ventilation, N (%)	47 (37)	18 (26)	29 (51)	0.049
Location of arterial catheter, N (%)				
Radial	113 (89)	67 (96)	46 (81)	0.007
Femoral	14 (11)	3 (4)	11 (19)	0.007
Left	54 (43)	28 (40)	26 (46)	0.5
Right	73 (57)	42 (60)	31 (54)	0.5
SOFA score, median (IQR)	8 (4–11)	5 (2–8)	11 (8.5–14.5)	< 0.001
Diagnoses, N (%)				
Bowel obstruction	5 (4)	3 (4)	2 (4)	0.8
Endocarditis	4 (3)	2 (3)	2 (4)	0.8
Incarcerated organs	4 (3)	4(6)	0 (0)	0.3
Ischemic organs	2 (2)	0 (0)	2 (4)	N/A
Liver failure	6 (5)	3 (4)	3 (5)	0.8
Pancreatitis	6 (5)	4(6)	2 (4)	0.6
Perforated viscus	12 (9)	4 (6)	8 (14)	0.1
Postoperative infection	11 (9)	6 (9)	5 (9)	0.9
Respiratory failure	9 (7)	7 (10)	2 (4)	0.2
Sepsis, unspecified	21 (17)	7 (10)	14 (57)	0.028
Soft tissue infection	46 (36)	29 (41)	17 (30)	0.2
Other	1 (1)	1 (1)	0 (0)	N/A
Time intervals between NIBP and IABP (minutes), median (IQR)	10 (0–15)	12 (0–16)	8 (0–11)	0.018
White blood cell counts (per microliter), mean (SD)	16.0 (10.8)	14.2 (9.7)	18.3 (11.7)	0.001
Serum lactate (mmol/L), mean (SD)	3.1 (3.1)	2.1 (1.8)	4.3 (3.9)	< 0.001
Hospital disposition, N (%)				
Discharge home	40 (32)	26 (37)	14 (25)	0.1
Acute rehabilitation facility	36 (28)	17(24)	19 (33)	0.3
Skilled nursing home	22 (17)	16 (23)	6 (11)	0.7
Dead/hospice	29 (23)	11 (16)	18 (32)	0.03

*BMI*, body mass index; *HTN*, hypertension; *CAD*, coronary artery disease; *PAD*, peripheral arterial disease; *IABP*, invasive arterial blood pressure; *NIBP*, non-invasive blood pressure; *mm Hg*, millimeters mercury; *PAD*, peripheral artery disease; *IQR*, interquartile range; *SOFA*, Sequential Organ Failure Assessment; *SD*, standard deviation; *mmol/L*, millimoles per liter.

**Table 2:** Comparison between blood pressure from IABP and NIBP monitoring modalities for septic patients. Patients requiring vasopressors had a greater likelihood of clinically significant discrepancy between IABP and NIBP compared to patients without vasopressor requirement. Arterial blood pressure monitoring was more likely to detect MAP ≤ 64 mm Hg among sepsis patients with vasopressors.

Variables	All patients (N = 127)	Without vasopressor (N = 70)	With vasopressor (N = 57)	P
Catheter-days (days), median [IQR]	3 [1–5]	2 [1–4]	4 [2–8.5]	<0.001
Type of vasopressor, N (%)^1^				
Norepinephrine	54 (43)	0 (0)	54 (95)	N/A
Epinephrine	11 (9)	0 (0)	11 (19)	N/A
Vasopressin	16 (13)	0 (0)	16 (28)	N/A
Mean arterial pressure of NIBP (mm Hg), mean (SD)	82 (19)	87 (20)	76 (16)	<0.001
Mean arterial pressure of IABP (mm Hg), mean (SD)	79 (19)	84 (19)	73 (16)	<0.001
Difference in Mean Arterial Pressure Between IABP and NIBP (mm Hg), mean (SD)	11 (12)	10 (10)	12 (15)	0.08
Number of patients MAP of NIBP ≤ 64 mm Hg, N (%)	12 (9)	5 (7)	7 (12)	0.3
Number of patients with a clinically significant discrepancy in MAP^2^	11 (9)	2 (3)	9 (16)3	0.01
Number of patients with MAP of IABP ≤ 64 mm Hg, N (%)^3^	25 (20)	6 (9)	19 (33)^4^	<0.001
Number of any complications, N (%)^5^	0 (0)	0 (0)	0 (0)	N/A

1 Patients were eligible to receive more than one vasopressor.

2 Clinically significant discrepancy was defined as Mean Arterial Pressure Difference ≥ 10 mm Hg and either NIBP’s or IABP’s reading was ≤ 64 mm Hg.

3 OR 6.4, 95% CI 1.2–30, P = 0.01.

4 OR 5.3, 95% CI 1.9–14.5, P < 0.001.

5 Complications from arterial catheters were defined as necrosis, source for blood stream infection, local infection, infiltration, bleeding, aneurysm. The 95% confidence interval (CI) for complications was 0 (95% CI 0–0.02).

*NIBP*, non-invasive blood pressure; *IABP*, invasive arterial blood pressure; *IQR*, interquartile range; *MAP*, mean arterial pressure; *mm Hg*, millimeters of mercury; *OR*, odds ratio; *SD*, standard deviation.

**Table 3:** Results from forward stepwise multivariable logistic regression measuring association between clinical factors and the likelihood of clinically significant discrepancy between NIBP and IABP*. All predetermined factors were entered into the models and only factors with significant association were reported. The models for each outcome measure showed both good fit of the independent variables and good discriminatory capability (higher AUROC**).

Variables	OR	95% CI	P	VIF
Outcome: Clinically Significant Blood Pressure Discrepancy^1^				
SOFA – each unit	1.33	1.02–1.73	0.034	2.0
Serum lactate – each mmol/L	1.27	1.003–1.60	0.047	2.1
Any kidney disease	0.03	0.002–0.51	0.015	2.6
Bowel obstruction	34	1.2–100+	0.035	1.4
Secondary outcome: MAP difference ≥ 10 mm Hg^2^				
SOFA – each unit	1.17	1.03–1.3	0.012	1.9
Peripheral artery disease	6.7	1.3–33.5	0.021	1.1
Incarcerated organs	16.4	1.4–100+	0.027	1.1

1 Homes-Lemeshow test chi-square 4.5, D(f) = 8; P = 0.81; AUROC: 0.92.

2 Homes-Lemeshow test chi-square 6.5, D(f) = 8, P = 0.59; AUROC: 0.72.

*AUROC*, area under the receiver operating characteristic curve; *OR*, odds ratio; *CI*, confidence interval; *D(f)*, degree of freedom; *mmol/L*, millimoles per liter; *IABP*, invasive arterial blood pressure; *NIBP*, non-invasive blood pressure; *SOFA*, Sequential Organ Failure Assessment score; *VIF*, variance inflation factor.

## References

[1] Rhodes A, Evans LE, Alhazzani W (2017). Surviving Sepsis Campaign: International Guidelines for Management of Sepsis and Septic Shock: 2016. Intensive Care Med.

[2] Li Y, Li H, Zhang D (2020). Timing of norepinephrine initiation in patients with septic shock: a systematic review and meta-analysis. Crit Care.

[3] Lakhal K, Macq C, Ehrmann S (2012). Noninvasive monitoring of blood pressure in the critically ill: reliability according to the cuff site (arm, thigh, or ankle). Crit Care Med.

[4] Gershengorn HB, Wunsch H, Scales DC (2014). Association between arterial catheter use and hospital mortality in intensive care units. JAMA Intern Med.

[5] Riley LE, Chen GJ, Latham HE (2017). Comparison of noninvasive blood pressure monitoring with invasive arterial pressure monitoring in medical ICU patients with septic shock. Blood Press Monit.

[6] Scalea TM, Rubinson L, Tran Q (2016). Critical care resuscitation unit: an innovative solution to expedite transfer of patients with time-sensitive critical illness. J Am Coll Surg.

[7] Keville MP, Gelmann D, Hollis G (2021). Arterial or cuff pressure: clinical predictors among patients in shock in a critical care resuscitation unit. Am J Emerg Med.

[8] Hiratzka LF, Bakris GL, Beckman JA (2010). 2010 ACCF/AHA/AATS/ACR/ASA/SCA/SCAI/SIR/STS/SVM Guidelines for the Diagnosis and Management of Patients with Thoracic Aortic Disease: a report of the American College of Cardiology Foundation/American Heart Association Task Force on Practice Guidelines, American Association for Thoracic Surgery, American College of Radiology, American Stroke Association, Society of Cardiovascular Anesthesiologists, Society for Cardiovascular Angiography and Interventions, Society of Interventional Radiology, Society of Thoracic Surgeons, and Society for Vascular Medicine. Circulation.

[9] Hemphill JC, Greenberg SM, Anderson CS (2015). Guidelines for the Management of Spontaneous Intracerebral Hemorrhage: A Guideline for Healthcare Professionals from the American Heart Association/American Stroke Association. Stroke.

[10] Dellinger RP, Levy MM, Carlet JM (2008). Surviving Sepsis Campaign: international guidelines for management of severe sepsis and septic shock: 2008. Crit Care Med.

[11] Worster A, Bledsoe RD, Cleve P (2005). Reassessing the methods of medical record review studies in emergency medicine research. Ann Emerg Med.

[12] Walsh M, Devereaux PJ, Garg AX (2013). Relationship between intraoperative mean arterial pressure and clinical outcomes after noncardiac surgery: toward an empirical definition of hypotension. Anesthesiology.

[13] Asfar P, Meziani F, Hamel J-F (2014). High versus low blood-pressure target in patients with septic shock. N Engl J Med.

[14] Garland A (2014). Arterial lines in the ICU: a call for rigorous controlled trials. Chest.

[15] Scheer BV, Perel A, Pfeiffer UJ (2002). Clinical review: complications and risk factors of peripheral arterial catheters used for haemodynamic monitoring in anaesthesia and intensive care medicine. Crit Care.

