# Supplementary material for: Discrepancy Between Invasive and Noninvasive Blood Pressure Measurements in Patients with Sepsis by Vasopressor Status
Source: West J Emerg Med. 2022 May 5;23(3):358–67. doi: 10.5811/westjem.2022.1.53211 (PMC9183768; doi:10.5811/westjem.2022.1.53211)
Supplement: Supplementary file 1 [file wjem-23-358-s001.docx]

**Appendix 1.** List of independent variables that were included in multivariable logistic regression that measured the association between clinical factors and clinically relevance of blood pressure monitoring of septic patients.  All variables were determined prior to analysis and were entered in the model in a forward stepwise manner.

| **Continuous variables** |
| --- |
| Age, each year |
| Body Mass Index -each unit |
| White Blood Cell count – each count per microliter  Serum Lactate level – each mmol/L  SOFA Score – each unit |
| **Categorical variables** |
| Gender (Male =1, Female =0) |
| Location of arterial catheter (Radial = 1, Femoral = 0) (Left = 1, Right = 0) |
| Past medical of Diabetes (Yes = 1, No =0)  Past medical history of Coronary Artery Disease (Yes =1, No =0) |
| Past medical history of Hypertension (Yes=1, No=0) |
| Past medical history of Peripheral Artery Disease (Yes=1, No=1) |
| Past medical history of Any Kidney Disease (Yes=1, No=0) |
| Receiving Mechanical Ventilation at time of measurements (Yes =1, No =0) |
| Diagnoses (Yes=1, No=0) |
| Bowel Obstruction     Endocarditis     Incarcerated Organs     Ischemic Organs     Liver Failure     Pancreatitis     Perforated Viscus     Post-Operative Infection     Respiratory Failure     Sepsis, unspecified     Soft Tissue Infection  Other |
| Receiving Any Vasopressors (Yes = 1, No =0)* |
|  |

*Vasopressor includes Norepinephrine, Epinephrine, and Vasopressin.

*mmol/L*, millimoles per liter; *SOFA,*Sequential Organ Failure Assessment
