# Supplementary material for: Discrepancy Between Invasive and Noninvasive Blood Pressure Measurements in Patients with Sepsis by Vasopressor Status
Source: West J Emerg Med. 2022 May 5;23(3):358–67. doi: 10.5811/westjem.2022.1.53211 (PMC9183768; doi:10.5811/westjem.2022.1.53211)
Supplement: Supplementary file 2 [file wjem-23-358-s002.docx]

**Appendix 2**. List of diagnoses (in alphabetical order) for included septic patients who were admitted to the critical care resuscitation unit.

| Diagnoses | N (%) |
| --- | --- |
| Bowel obstruction | 5 (4) |
| Endocarditis | 4 (3) |
| Incarcerated organs | 4 (3) |
| Ischemic organs | 2 (2) |
| Liver failure | 6 (5) |
| Pancreatitis | 6 (5) |
| Perforated viscus | 12 (9) |
| Postoperative infection | 11 (8) |
| Respiratory failure | 9 (7) |
| Sepsis | 21 (17) |
| Soft tissue infection | 46 (36) |
| Other | 1 (1) |
